# Cow’s Milk and Immune Function in the Respiratory Tract: Potential Mechanisms

**DOI:** 10.3389/fimmu.2018.00143

**Published:** 2018-02-12

**Authors:** Olaf Perdijk, Marloes van Splunter, Huub F. J. Savelkoul, Sylvia Brugman, R. J. Joost van Neerven

**Affiliations:** ^1^Cell Biology and Immunology Group, Wageningen University & Research, Wageningen, Netherlands; ^2^FrieslandCampina, Amersfoort, Netherlands

**Keywords:** raw cow’s milk, upper airways, respiratory syncytial virus, allergies, asthma, barrier functioning, immune regulation, gut–lung axis

## Abstract

During the last decades, the world has witnessed a dramatic increase in allergy prevalence. Epidemiological evidence shows that growing up on a farm is a protective factor, which is partly explained by the consumption of raw cow’s milk. Indeed, recent studies show inverse associations between raw cow’s milk consumption in early life and asthma, hay fever, and rhinitis. A similar association of raw cow’s milk consumption with respiratory tract infections is recently found. In line with these findings, controlled studies in infants with milk components such as lactoferrin, milk fat globule membrane, and colostrum IgG have shown to reduce respiratory infections. However, for ethical reasons, it is not possible to conduct controlled studies with raw cow’s milk in infants, so formal proof is lacking to date. Because viral respiratory tract infections and aeroallergen exposure in children may be causally linked to the development of asthma, it is of interest to investigate whether cow’s milk components can modulate human immune function in the respiratory tract and *via* which mechanisms. Inhaled allergens and viruses trigger local immune responses in the upper airways in both nasal and oral lymphoid tissue. The components present in raw cow’s milk are able to promote a local microenvironment in which mucosal immune responses are modified and the epithelial barrier is enforced. In addition, such responses may also be triggered in the gut after exposure to allergens and viruses in the nasal cavity that become available in the GI tract after swallowing. However, these immune cells that come into contact with cow’s milk components in the gut must recirculate into the blood and home to the (upper and lower) respiratory tract to regulate immune responses locally. Expression of the tissue homing-associated markers α4β7 and CCR9 or CCR10 on lymphocytes can be influenced by vitamin A and vitamin D3, respectively. Since both vitamins are present in milk, we speculate that raw milk may influence homing of lymphocytes to the upper respiratory tract. This review focuses on potential mechanisms *via* which cow’s milk or its components can influence immune function in the intestine and the upper respiratory tract. Unraveling these complex mechanisms may contribute to the development of novel dietary approaches in allergy and asthma prevention.

## Introduction

In the Western world, the prevalence of chronic inflammatory diseases, including allergies, has increased dramatically in the last few decades, while the number of serious infectious diseases has declined rapidly (1). An inverse correlation, indicating a “protective effect” of infectious diseases against chronic inflammatory diseases (e.g., allergy and asthma), was postulated in 1989 by Strachan, who formulated the hygiene hypothesis (2). The hygiene hypothesis suggests that the exposure to viruses and bacteria is essential to induce a T-helper (Th)1 response, which balances the immune system and protects against Th2-mediated diseases. With the discovery of additional T cell subsets such as Th17 cells and regulatory T cells (Tregs), this paradigm had to be revised. For example, it was demonstrated that suppressive dendritic cells (DCs) induced by helminths restored the disturbed Th1/Th2 balance by induction of Tregs (3). The immune education of DCs was suggested to be an important step toward understanding the complex relation between infectious diseases and allergies (4). Th2 responses are now known to be enhanced by the production of type 2 cytokines (e.g., TSLP, IL-25, IL-33) secreted by group 2 innate lymphoid cells and epithelial cells (5, 6). Thus, different cell types are responsible for Th2-mediated diseases such as allergies. Allergy is initiated as an aberrant immune response towards a harmless antigen (allergen). *Via* activation of Th2 cells, the allergen triggers the production of allergen-specific IgE by B cells that binds to high-affinity FcεR1 on effector cells like mast cells and basophils. Effector cells release soluble factors (e.g. histamine) upon secondary exposure to the allergen that cause immediate type I allergic symptoms. The term “atopic march” refers to the sequence of IgE responses and clinical symptoms initiated in early life (7).

In the first year of life, the mucosal immune system is shaped by microbial colonization and dietary components, which contributes to health later in life (8). Viral infections during this critical period also impact health later in life. For example, respiratory syncytial virus (RSV) infection in early life was shown to increase the risk of wheezing up to 11 years of age (9) and allergic sensitization and development of asthma into adulthood (10–12). The exact mechanism by which allergy and viral infection in the upper airways results in the development of asthma is not yet elucidated. However, Holt and Sly (13) proposed a mechanism in which viral infection can trigger excessive type I interferon production that can result in upregulation of FcεR1 expression on airway resident DCs. FcεR1-mediated signaling in DCs has been suggested to contribute to allergic airway inflammation depending on the environmental stimuli (14). In mice, cross-linking of virus-specific IgE on these airway DCs results in the production of Th2 cytokines and the chemoattractant CCL28, recruiting effector Th2 cells to the airways (15, 16). However, recent evidence shows that not all asthma patients have this typical Th2 profile in early life (17). Nevertheless, atopy and viral infections in early life are risk factors for asthma development. Therefore, preventive strategies for asthma, such as dietary interventions, should be targeted at early life to suppress allergen- or viral-induced airway inflammation.

In Europe, rapid evolutionary changes are found in the lactase persistance gene suggesting health benefits of cow’s milk consumption to humans (Box 1). The existing epidemiological evidence shows that consumption of cow’s milk in early life is associated with a lower prevalence of allergies, respiratory tract infections, and asthma. This suggests that milk components (e.g., proteins, sialylated oligosaccharides, and vitamins) may contribute to the protection against the development of allergies (18) and respiratory viral infections (19). Since raw cow’s milk may contain pathogenic bacteria, intervention studies in infants are impossible due to safety risks. Nevertheless, a recent mouse study showed a causal relation between raw milk consumption and the protection against house dust mite (HDM)-induced asthma, which was not seen in mice receiving heated milk (20). The mechanisms underlying this protective effect of raw cow’s milk remains speculative. Therefore, in this review we discuss potential mechanisms by which dietary components, using cow’s milk as an example, can protect against airway inflammation.

Box 1 The consumption of cow’s milk in Europe and the Middle East already dates back to the Neolithic cultural period. Milk fatty acids were traced by carbon isotope analysis on Middle Eastern pottery, showing that cow’s milk was already processed since 6500 BC (154). This introduction of processed ruminant milk might explain why it was adopted so quickly, despite lactose intolerance. Lactase persistence (i.e., the capacity to digest lactose into adulthood) seems to have arisen around 5500 BC in the European population due to a specific mutation in the gene encoding the lactase enzyme (155). Its rapid expansion in the ancestral population suggests high selective pressure (156). This makes it appealing to speculate that this mutation confers health benefits to the host by consuming cow’s milk.

## Homology Between Cow’s Milk and Breast Milk

By comparing the immunomodulatory components in breast milk with those in cow’s milk, conserved mechanisms could be identified, which contribute to immune homeostasis in early life. Overall bovine and human milk contain similar components. However, the concentration or presence of several specific components (e.g., β-lactoglobulin specific for cow’s milk) may differ. For a complete overview comparing breast milk and cow’s milk, we refer to a review by van Neerven et al. who compared the composition of breast milk to cow’s milk (18). We briefly describe several immunomodulatory components in cow’s milk that are used in this review to illustrate potential mechanisms by which cow’s milk may affect respiratory health.

Systematic reviews conclude that TGFβ consumption in early life protects against allergies in humans and animal models (21, 22). Strikingly, the active forms of TGFβ1 and TGFβ2 are identical between cow and human (23). Although articles report different concentrations of TGFβ in breast and cow’s milk, there is a consensus that TGFβ2 is several fold more abundant compared to TGFβ1 (21, 24). Remarkably, the concentrations of TGFβ1 and TGFβ2 are approximately fivefold more abundant in cow’s milk compared to breast milk (18). The concentration of TGFβ1 in cow’s milk decline significantly after processing and are non-detectable in processed milk (25).

Bovine lactoferrin has 77% homology to human lactoferrin on mRNA level and 69% on protein level (26). Nevertheless, bovine lactoferrin is taken up by the human lactoferrin receptor and exerts similar bioactivities as human lactoferrin on human colon epithelial cells such as induction of proliferation, differentiation, and TGFβ expression (27). Similarly, bovine IL-10 is 76.8% homologous and affects human cells (28). Cow’s milk shows lower IgA and higher IgG levels compared to human milk (18).

While the quantities of proteins in human milk are quite similar to cow’s milk, the oligosaccharide composition is completely different. In contrast to cow’s milk, human milk is unique among mammals in its high and diverse levels of complex oligosaccharides (29). Cow’s milk contains only small amounts and a non-diverse profile of oligosaccharides, which is dominated by sialylated oligosaccharides. Therefore, this review will only address the effect of sialylated oligosaccharides present in cow’s milk. In colostrum, the concentrations of sialylated oligosaccharides range between 0.23–1.5 and 1–3.3g/L in cows and humans, respectively (30). The concentrations of sialylated oligosaccharides in mature bovine milk are approximately 10-fold lower compared to colostrum (30). Most sialylated oligosaccharides in human- and cow’s milk are monomeric [e.g., 3′-sialyllactose (3′SL) and 6′-sialyllactose (6′SL)] and are present in very low concentrations in infant formulas (31).

Vitamin A and D, which are not specific for cow’s milk, are essential for the development of the mucosal immune system. Vitamin A can be obtained from different dietary sources and can be converted to its active metabolite retinoic acid (RA) by epithelial cells and DCs in the gut (32). Breast milk contains, depending on the vitamin D status of the mother, low levels of vitamin D3, and additional vitamin D3 supplementation is recommended for infants (33). Cow’s milk contains similar concentrations of vitamin A, vitamin D3, and 1,25-hydroxyvitamin D3 (active form of vitamin D3) compared to breast milk (18, 34).

## Epidemiological Evidence for the Immune Modulatory Role of Cow’s Milk on Respiratory Health

It is now well established that children growing up on a farm less often develop allergies and asthma (35). Of the different environmental factors investigated in these epidemiological studies, contact with farm animals, endotoxin levels in house dust, and the consumption of farm milk (i.e., cow’s milk with an unknown heating status) showed the strongest association with the protection of childhood asthma and allergy (35, 36). The consumption of farm milk was associated with higher Treg numbers in blood, which were negatively associated with asthma and serum IgE levels (37). Moreover, increased demethylation of the *FOXP3* gene and increased FoxP3^+^ T cell numbers were detected in PBMC cultures of children who were exposed to farm milk, suggesting that farm milk consumption induces an immunoregulatory phenotype.

Raw cow’s milk consumption in the first year of life showed an inverse correlation with the prevalence of atopy and doctors-diagnosed asthma in farmers and non-farmers (38). This study showed that raw cow’s milk consumption in the first year of life is inversely associated with atopic sensitization and asthma independently of the farming environment. Children who consumed raw cow’s milk produced higher IFN-γ levels upon whole blood stimulation (39). Since IFN-γ production is associated with a Th1 profile, this finding—even though counterbalancing Th2 responses—is in contrast to studies showing a regulatory phenotype induced by farm milk consumption in the first year of life (37). Nevertheless, both studies show that either raw cow’s milk or farm milk is associated with lower total serum IgE levels and allergic diseases (37, 39). Other epidemiological studies have specifically addressed the question whether heating of farm milk influences its effect on allergic diseases.

Loss et al. showed that the protective effect of cow’s milk on asthma and hay fever incidence was only noted in children who consumed raw milk and not in children who consumed high heat-treated shop milk (>85°C). Indeed, the thermosensitive whey proteins BSA, α-lactalbumin, and β-lactoglobulin were associated with the protective effects. Similar trends were found for lactoferrin and total IgG. No associations were found between microbiological communities or cell counts in the milk, showing that the protective effect was not primarily caused by bacteria in the raw cow’s milk (40). A follow-up study investigated the association between raw, boiled, or commercially available cow’s milk consumption and the occurrence of common infections in infants (2–12 months of age). In comparison to ultra-heat-treated milk, raw milk consumption in the first year of life was inversely associated with the occurrence of rhinitis, otitis, and respiratory tract infections at 12 months of age. In addition, soluble CRP levels were lower in the infants that received raw cow’s milk. Interestingly, respiratory tract infections and fever were also reduced in infants receiving boiled cow’s milk (19). It was suggested that the milk fat globule membrane contributes to this negative association between boiled milk consumption and respiratory tract infections (19). Indeed, non-heat-sensitive cow’s milk components may also contribute to the induction of a regulatory phenotype (37). Nevertheless, these studies show that the thermosensitive fraction of the milk (i.e., proteins, most likely whey fraction) is an important driver of the protection against not only allergies and asthma but also viral infections, fever, and inflammatory conditions in the upper airways.

These epidemiological findings cannot be confirmed in controlled intervention studies in infants due to safety risks. However, controlled trails with infants fed experimental infant formulas rich in immune-related bovine milk components have shown effects on respiratory tract infections. Infants fed with a bovine milk fat globule membrane preparation rich in IgG and lactoferrin showed a reduced prevalence of acute otitis media and showed lower pneumococcal-specific IgG levels in serum (41). Similarly, infants of 4–6 months of age receiving infant formula supplemented with lactoferrin showed fewer respiratory illness (42, 43). A reduction in respiratory tract infections was also observed in an intervention study with children of 1–6 years of age receiving bovine colostrum that is extremely rich in IgG (44). These findings indicate that bovine milk components may prevent respiratory tract infections in early life.

## Passage Through the Gastrointestinal Tract

After swallowing milk components, allergens, or pathogens, they pass through the GI tract and are exposed to different pH levels and proteases, varying from pancreatic, gastric, or peptidases on the enterocytic brush border. In adults, there is little evidence that intact dietary proteins can reach the circulation in homeostasis (45). In early life, however, the digestion of proteins is lower compared to adults, which has several causes. First, the gastric acid production in infants only reaches the levels of adults after 6 months of age. Infants therefore have a higher pH in the stomach compared to adults. Lower gastric acid levels impair the activity of pepsins. Second, concentrations of other proteases (e.g., chymotrypsin and enterokinase) are significantly lower in the small intestine of neonates (10–60% of that of adults) compared to adults (46). Thus, in infants, a significant fraction of milk proteins reaches the small intestine intact and may interact with intestinal immune cells (e.g., epithelial cells and sampling DC). For instance, 10% of the orally fed bovine IgG (bIgG) can be found in stool of infants, compared to <0.1% in adults (47). In addition, the infants gut is in a “leaky state” (48), which may promote sensitization to allergens and bacteria- or virus-induced inflammation. On the other hand, it is a window in which (milk-derived) components have an opportunity to induce tolerance.

Some milk proteins are less sensitive to the low pH and proteases and pass the GI tract intact or can even be activated by an acidic environment or protease activity. For instance, TGFβ, which is present in milk in its latent form first needs to be activated before exerting any effector function. The activation of this exogenous latent TGFβ can be triggered by multiple factors such as macrophages membrane-bound receptor TSP-1, αvβ-3/5/6 and αvβ8 integrins, ROS, low pH during passage of the stomach, and proteases (49, 50). Thus, TGFβ can be activated by binding integrins in the upper airways or by activation in the stomach and small intestine. Significant amounts of the abundant milk protein lactoferrin reach the small intestine intact and retain their functional activity in both adults and infants (26).

In contrast to proteins, milk oligosaccharides escape enzymatic hydrolysis in the small intestine and low pH of the stomach and are fermented in the colon (51). By escaping degradation in the small intestine, they function as a carbon source for the microbiota in the colon and can be converted into metabolites such as short-chain fatty acids (SCFAs). In breastfed infants, the genus *Bifidobacterium* is commonly present, which comprises mainly *Bifidobacterium bifidum, Bifidobacterium longum* subsp. *infantis*, and *Bifidobacterium breve*. Of the three, *B. longum infantis* has the right machinery to ferment sialylated oligosaccharides directly (52, 53) and is unique in its ability to import and degrade low-molecular-weight oligosaccharides (54). Indeed, several *B. longum* strains were capable of converting 3′SL and 6′SL, which are abundantly present in bovine milk, into SCFA *in vitro* (55). Nevertheless, it is unknown whether the concentrations of sialyllactose present in cow’s milk alters the microbiota *in vivo*. *B. longum* is abundantly present in breast-fed neonates and is thought to confer various health benefits (e.g., enhanced barrier functioning and anti-inflammatory effects) to the host (56). Although most of these sialylated oligosaccharides are fermented by these *Bifidobacteria*, a small fraction of oligosaccharides reaches the circulation intact (57, 58). Therefore, sialylated oligosaccharides might impact immunity directly. Interestingly, it has been shown that the microbial community in the upper respiratory tract can be differentially modulated by breast milk compared to formula-fed children (59). Interestingly, breastfed infants showed a higher prevalence of *Dolosigranulum* that was negatively associated with respiratory tract infections (59). Children with asthma show a lower nasal microbiota composition and higher abundance of *Moraxella* (60). *Moraxella* was not associated with asthma in children who were exposed to a farming environment, which was in contrast to children who were not exposed to a farming environment. This indicates that the farming environment might protect the children from the detrimental effects of *Moraxella*. To date, it is unknown whether raw cow’s milk alters the nasopharyngeal microbiota composition and if this influences susceptibility toward upper respiratory tract infections or allergies.

## Binding of Bovine IgG to Respiratory Pathogens

One mechanism by which food components could modulate immunity in the (upper) respiratory tract is by preventing contact between pathogens or allergens and the host immune system. In early life, maternal antibodies are essential for passive protection of the infant against viral infections. Interestingly, maternal RSV-specific antibodies in amniotic fluid were recently shown to protect mouse pups from RSV infection for at least 1 week after birth (61). Human antibodies are found against conserved parts of the pre-fusion F protein of human RSV and metapneumovirus (PMV) that cross-neutralize bovine RSV (62). This cross-reactivity could also work *vice versa* if bovine IgG could recognize conserved patterns on human RSV. Cross-reactive antibodies to other human pathogens have also been demonstrated in bovine milk and colostrum. Indeed, as reviewed by van Neerven, feeding colostrum of cows vaccinated against specific human pathogens protected children from subsequent infections (63). Interestingly, bovine IgG was shown to bind human RSV and to induce phagocytosis *via* FcγRII receptors on macrophages, neutrophils, and monocytes (64). The binding of bovine IgG to RSV also directly neutralizes RSV, as shown by protection of Hep2 cells from infection with RSV *in vitro* (64). Bovine IgG isolated from cow’s milk does not only bind to human viruses but was also found to bind to inhaled allergens (e.g., HDM) (65). In addition, bovine IgG inhibits translocation of Pam3CSK4 over the epithelial barrier, thereby suppressing the production of pro-inflammatory cytokines *in vitro* (66). Thus, bovine IgG can neutralize RSV infection *in vitro* and might also play a role in preventing sensitization by binding allergens and supporting barrier functioning by preventing binding of TLR ligands to the epithelium.

**Figure 1:**
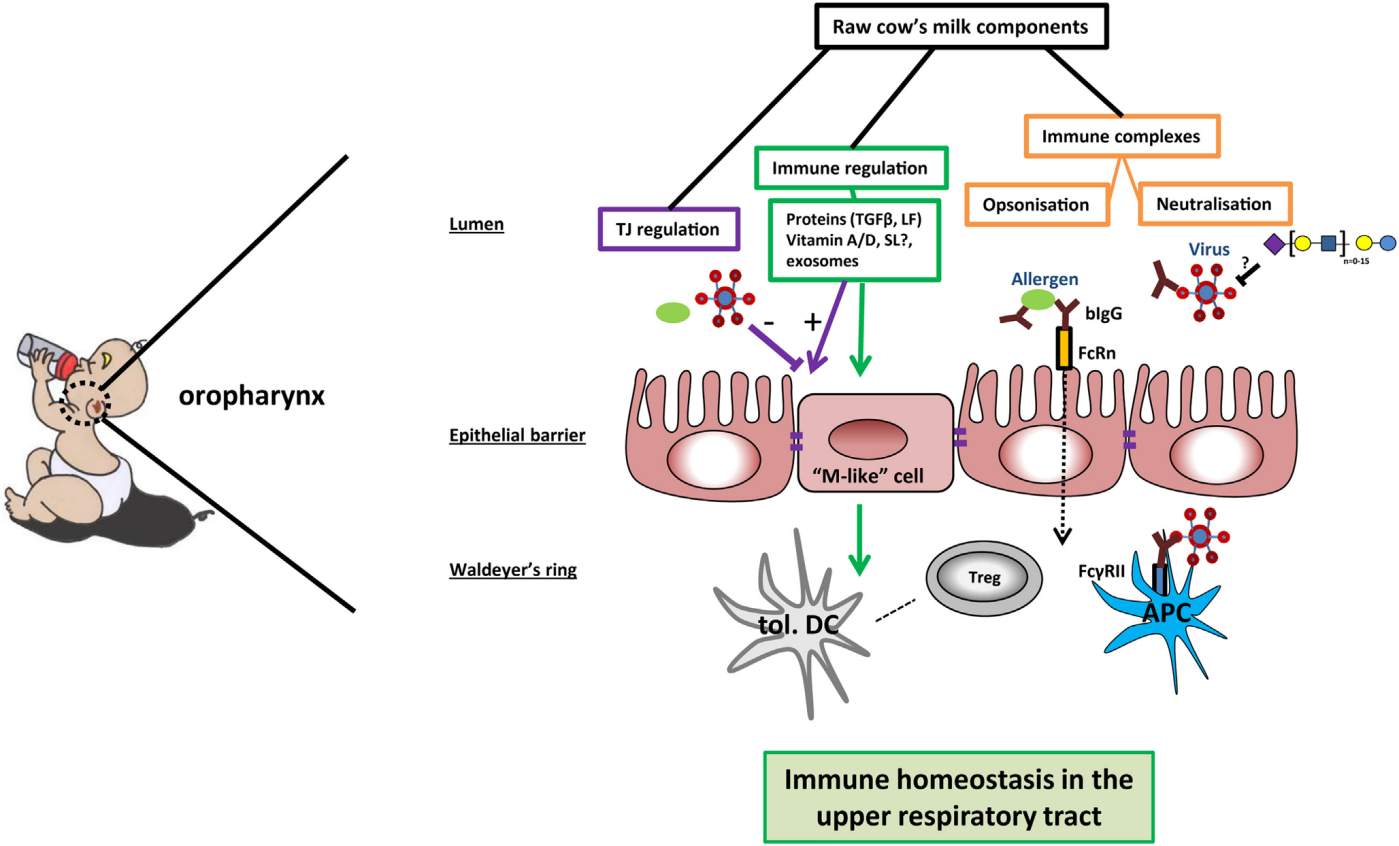
Potential mechanisms of cow’s milk-induced immune homeostasis in the upper respiratory tract. In the oropharynx, raw cow’s milk components can contribute to immune homeostasis *via* different mechanisms. First, bovine IgG can bind and possibly even neutralize bacteria, viruses, or allergens. Immune complexes are transported over the epithelial barrier by neonatal Fc receptor (FcRn) or transported *via* M-like cells to reach the mucosal tissue in the Waldeyer’s ring. The immune complexes can bind to FcγRII on antigen-presenting cells (APCs), leading to phagocytosis and clearance of the pathogens—as well as antigen presentation to (regulatory) T cells. Second, sialylated oligosaccharides may function as decoy receptors for viruses in the lumen of the oropharynx, preventing viral adhesion. Further, the expression of tight junction proteins can be enhanced by several milk components, thus strengthening the mucosal barrier against breaching by allergens and pathogens. Finally, several milk components contribute to immune regulation by inducing the differentiation into tolerogenic dendritic cells (tol. DC) and immunoregulatory T cells (Tregs). In this way, raw cow’s milk can promote a local microenvironment that contributes to immune homeostasis in the upper airways.

Next to bovine IgG, milk oligosaccharides have also been shown to prevent binding of viruses to host cells (29). Viruses use lectin-like structures to adhere and infect host cells. It was hypothesized that breast-fed infants developed less otitis caused by viral infections (e.g., RSV and influenza) due to the decoy receptor activity of milk oligosaccharides (29). However, as stated in the review by ten Bruggencate et al., it is to date uncertain which sialylated oligosaccharides can serve as a decoy receptor for human respiratory infecting viruses (30). Thus, IgG and sialylated oligosaccharides present in cow’s milk might shield allergens or virus pathogens from inducing infection and inflammation (Figure 1).

## The First Host Barrier: The Epithelium

When the allergen or virus is not neutralized, it will encounter the epithelial cell layer of mucosal tissues. This epithelial cell layer is the first line of defense in mucosal tissues. Epithelial cells are covered by a thick layer of mucus that keeps harmful compounds from entering the body. Epithelial cells act as the first physical barrier and the first responders of the innate immune system. The epithelial cells protect against inflammation and sensitization by preventing bacteria and virus entry and leakage of allergens into the mucosal tissue. The integrity of the epithelial barrier in the upper airways (67) and intestine (68) is regulated by tight junctions (TJs). Homeostasis is maintained by hyporesponsiveness of epithelial cell toward bacterial constituents, because inflammation disrupts barrier functioning (69). Therefore, the barrier functioning of the upper airways is under constant threat of environmental factors, including viral infections (e.g., RSV) and allergens (70). Breaching of the barrier can results in tissue modifications in the upper airways as seen in patients suffering from allergic rhinitis or sinusitis (71).

### Tight Junctions

The integrity of the epithelial barrier is maintained by structural elements including adherens junctions, desmosomes, and TJs. TJs consists of a “ziplock-like” structure of multiple protein strands that are connected to the cytoskeleton, allowing selective transport across the barrier (72). Cytosolic scavenger proteins (e.g., ZO-1) link the actin cytoskeleton to paracellular located proteins: the claudins and occludins. The claudin family consists of transmembrane proteins, which *via* the interaction of claudin strands, are connected to each other by extracellular loops (73). Claudins can be subdivided into pore-forming (e.g., claudin-2) and sealing claudins (e.g., claudin-4) that increase and decrease permeability, respectively (73). Notably, pro-inflammatory cytokines (e.g., IL-6 and TNF) increase the expression of the pore-forming claudin-2 and thereby reduce the epithelial barrier function (74). The function and distribution of occludins is highly influenced by its phosphorylation status that is regulated by protein kinase C (75). TJ proteins are key in maintaining epithelial barrier function and are shown to regulate proliferation on gene expression level (76). For further reading about TJ proteins, we would like to refer to other excellent reviews specifically about occludins (75), claudins (73), or the function and morphology of TJs in general (72, 77).

Many allergens have protease activity that could breach barrier functioning (78). For instance, the protease activity of one of the major HDM allergens, Der p 1, was shown to disrupt the cleavage site in the extracellular loops of claudins and occludins (79). In contrast, RSV disrupts barrier functioning by remodeling the actin cytoskeleton and interfering with cytosolic scavenger proteins (80). Disruption of barrier functioning in the upper airways results in increased exposure of allergens and viral particles to the underlying immune system (Figure 1), which could result in chronic inflammatory diseases such as asthma and allergies (81). In early life, the epithelial barrier is more permeable compared to adults. Closure of the barrier occurs only after a few weeks in humans, while in mice, this is a more gradual process that develops during weaning (48, 82).

### Effect of Milk Components on Barrier Functioning

Breast milk contains many growth factors that facilitate gut maturation. Neonates receiving infant formulas were shown to have a higher gut permeability compared to breast-fed neonates. This stresses the importance of identifying functional milk components that promote barrier functioning (48). Currently, there is no *in vivo* evidence on effects of cow’s milk on epithelial barrier functioning. Nevertheless, at least two recent studies investigated the effect of cow’s milk on epithelial cells *in vitro*. To study barrier functioning of dietary components *in vitro*, most studies use colon carcinoma cell lines (Caco-2 or HT-29). It is to date impossible to study the effect of dietary components on barrier functioning in the upper airways since no human oropharyngeal epithelial cell lines are available that form TJs. Caco-2 cells express enzymes that are expressed in the fetal intestine and are biochemically and morphologically similar to ileal enterocytes (83). These *in vitro* models are thus one of the few limited models available to study the effect of dietary components on TJ regulation. The anti-inflammatory properties of milk components on the epithelium are reviewed by Chatterton et al. (23). In this review, the role of dairy components on epithelial barrier function in terms of epithelial proliferation, differentiation, and TJ regulation is addressed.

The first study that looked at the effect of cow’s milk *in vitro* showed that cow’s milk induces the expression of the pore-forming TJ protein claudin-2 in Caco-2 cells (84). However, no differences were observed in permeability, which was proposed to be counteracted by the milk-induced increase of endogenous TGFβ expression. A second study stimulated HT-29 cells with raw milk versus pasteurized cow’s milk preparations. The authors showed with microarray analysis that raw milk induced the expression of genes related to immunity compared to the pasteurized cow’s milk or medium control (85). This study showed that the thermosensitive milk fraction (i.e., proteins) induced the expression of immune-related pathways and thereby indirectly barrier functioning.

One of the proteins in cow’s milk that is important for epithelial barrier functioning is TGFβ. Apart from these exogenous sources of TGFβ, TGFβ is endogenously produced. In the gut, TGFβ is most prominently expressed in epithelial cells compared to its expression in the underlying lamina propria (86). TGFβ1 is capable of promoting barrier functioning by regulating TJ expression and proliferation. TGFβ1 induces the expression of claudin-4 and protein kinase C expression *in vitro*, both strengthening the barrier (87, 88). On the other hand, TGFβ inhibits the proliferation of epithelial cells (86, 89). Interestingly, the production of endogenous TGFβ1 by epithelial cells is regulated through a positive feedback loop by other milk proteins like lactoferrin that triggers an intracellular cascade that results in the production of TGFβ1. Bovine and human lactoferrin were shown to have similar effects on barrier functioning. Moreover, in low concentrations, lactoferrin induces differentiation of epithelial cells, whereas lactoferrin stimulates proliferation in higher concentrations (26).

In high concentrations, sialylated milk oligosaccharides affect the cell cycle and induce differentiation of intestinal epithelial cells (90). In the colon, these oligosaccharides are fermented by the microbiota. These microbes produce SCFAs that also impact barrier functioning. As reviewed by Tan et al., SCFAs reduce paracellular permeability and induces the expression of TJ genes and MUC2 expression, thus strengthening the epithelial barrier (91) that may subsequently protect the host against infections (92).

Another milk ingredient shown to have immunomodulatory effects is vitamin D. More specifically, the inactive and circulating form of vitamin D3 (25(OH)2D3) is converted to the active form (1,25(OH)2D3) by the enzyme 1α-hydroxylase, which is highly expressed in the kidney and lowly expressed in epithelial cells (93). Epithelial cells transport the inactive form of vitamin D3 over the membrane, which can be subsequently systemically metabolized (94). The conversion locally by epithelial cells of dietary inactive vitamin D3 into the active form can create a microenvironment containing active vitamin D3. Stimulation of Caco-2 cells with 1,25(OH)2D3 was shown to result in the induction of E-cadherin, which indirectly promotes the transcription of ZO-1 and induces differentiation (95). In support of this, blocking vitamin D receptor transcription resulted in a decreased transepithelial electrical resistance and expression of ZO-1 and E-cadherin and claudin 1, 2, and 5 but not occludin (96). Thus, it is evident that vitamin D3 contributes to epithelial barrier function by regulating TJ protein expression. Less is known about the effect of vitamin A on barrier functioning. RA was shown to enhance differentiation of epithelial cells, as indicated by the increase in alkaline phosphatase expression. In contrast, RA also decreased the expression of claudin-2, resulting in a decrease in permeability of the Caco-2 model (97). Thus, several components present in cow’s milk promote epithelial barrier functioning (Figure 1).

## Do Milk Components Promote Immune Homeostasis?

The nasal mucus is cleared to the back of the throat every 10–15 minutes by the movement of cillia. Thus, it is likely that allergens and viruses are trapped in this thick layer of mucus and are subsequently swallowed. The oropharynx (throat) is the place where milk components, bacteria, viruses, and allergens may interact before they are digested. Lymphoid tissues in the upper airways are the lingual tonsils, tubal tonsils, palatine tonsils, and adenoids, together forming the Waldeyer’s ring (98). Uptake of antigens by the tonsils occurs *via* M-like cells in specialized induction sites, which are composed of follicles containing both myeloid and lymphoid cells (99). Similarly, in the GI tract, antigens can be taken up by columnar epithelial cells (transcellular), M cells, neonatal Fc receptor-mediated uptake (100), or direct uptake by specific sampling subsets of DCs (101).

The mucosal immune system is capable of distinguishing between harmful and harmless compounds resulting in inflammation or tolerance, respectively. Food components are important non-self-antigens to which an immune response constantly needs to be suppressed. This type of tolerance induction is known as oral tolerance. Food does not only trigger local tolerance but also systemic tolerance, and thus food makes the systemic and mucosal immune systems relatively unresponsive to these food antigens. Breast milk contains many components that dampen immune responses. It is suggested that this regulatory milieu induced to breast milk components favors tolerance inductions towards other harmless antigens such as allergens (102). This suppression of immune responses is antigen specific and long lasting.

The consumption of farm milk is associated with higher regulatory FoxP3^+^ T cell numbers, which were negatively associated with doctors-diagnosed asthma and IgE levels (37). We here address several potential cow’s milk components that might promote these regulatory responses. Literature supports that raw cow’s milk contains a multitude of components, including proteins and vitamins, that promote the development of human “tolerogenic” or regulatory monocyte-derived DCs (moDCs) *in vitro*.

Cow’s milk and colostrum contain several immunoregulatory cytokines such as TGFβ and IL-10. Interestingly, a population of tolerogenic IL-10 producing DCs (IL-10 DCs) with similar characteristics to *in vitro* monocyte-derived DCs, differentiated in the presence of IL-10, were identified in human blood (103, 104). Not only human IL-10 but also bovine IL-10, which has 70% homology to human IL-10, was shown to induce a dose-dependent reduction of CD80/CD86 expression and IL-12 and TNF production (28). DCs with low CD86/CD80 expression in the presence of TGFβ or IL-10 are known to polarize naive T cells into FoxP3^+^ T cells (105). IL-10 DCs also express PD-L1 which is critical for the induction of T cell anergy. Similarly, moDC differentiated in the presence of bovine lactoferrin showed inhibited cytokine responses and surface marker expression upon stimulation with TLR ligands (106).

TGFβ is an unique pleiotropic cytokine that is produced by leukocytes and epithelial cells (107). The dual role of TGFβ was shown in a recent review, which showed that TGFβ-induced SMAD proteins are key in balancing immunity (108). DCs from the lamina propria are essential for inducing FoxP3 expression in naive T cells, which requires an exogenous source of TGFβ (109). Interestingly, pups of mice exposed to airborne allergens developed oral tolerance towards the allergen that was dependent on milk-derived TGFβ (110). In addition, TGFβ and IL-10 inhibit type I interferon production by pDCs (111). Immunosuppressive cytokines such as IL-10 and TGFβ in milk are important in maintaining immune homeostasis and the suppression of type I interferon production. These immunosuppressive cytokines in cow’s milk could be essential for inducing a regulatory milieu, which subsequently may results in tolerance towards allergens.

Antigen-specific IgG in breast milk was shown to protect against OVA-induced asthma in a mouse model by inducing regulatory responses. Moreover, pups of mothers that were exposed to antigen aerosols during lactation resulted in a regulatory immune response that protected them from developing asthma (112). The proposed mechanism involves binding of IgG to neonatal Fc receptor (FcRn), which resulted in the expansion of antigen-specific Tregs. Bovine IgG shows some affinity for human FcRn (113) and is specific for human allergens (65), and it is therefore possible that the uptake of bovine IgG–allergen complexes induces FoxP3^+^ T cells (Figure 1). These functional properties of milk proteins are lost upon heating. Another heat-sensitive fraction of bovine milk that has been suggested to induce immune regulation is exosomal microRNA (114).

The role of milk oligosaccharides in the induction of oral tolerance remains inconclusive. 6′SL was shown to alleviate OVA-induced food allergic symptoms by promoting IL-10-producing T cells (115). In contrast, pups fed milk that contained 3′SL had more severe induced colitis compared to pups fed milk devoid of 3′SL. *Ex vivo* cultures of mesenteric lymph node (MLN) DC showed direct TLR4 activation by 3′SL (116). However, 3’SL did not induce TLR4-mediated activation of human immune cells *ex vivo* (117). In addition, sialylated milk oligosaccharides were shown to alter the microbiota composition and growth in infants (118). These changes in microbiota composition in turn impact the production of SCFAs that were shown to be essential, together with vitamin A, in oral tolerance induction (119). In summary, the direct immunomodulatory effect of sialylated oligosaccharides remains inconclusive. Rather than having direct effect on the immune system, sialylated oligosaccharides may promote immune homeostasis indirectly by promoting the outgrowth of SCFA-producing bacteria.

In the gut, a subset of migratory DC expressing the integrin CD103 are known to convert vitamin A into RA. In mouse models, RA induces the differentiation of naive T cells into Tregs *in vivo* (120). These findings were confirmed in vitro in humans by differentiating moDC in the presence of RA. These RA DCs expressed CD103 and were capable of polarizing naive T cells into Tregs (121) or FoxP3^−^ IL-10-producing T cells (122). Similarly, under steady-state conditions, lung macrophages produce RA and TGFβ toward harmless airborne antigens and induce antigen-specific Tregs (123). Thus, dietary vitamin A triggers endogenous RA production that is essential to induce Tregs in the gut and the lung. However, it is unknown whether dietary vitamin A contributes in the upper airways to induce antigen-specific Tregs. The active form of vitamin D3, 1,25(OH)2D3, halts the differentiation of monocytes into moDC *in vitro* and does not affect pDCs (124). These vitamin D3 DCs are less sensitive to TLR ligands and develop a semi-mature phenotype upon stimulation. These authors show that this semi-mature phenotype is instrumental for priming naive T cells to become Tregs and to induce T cell anergy (124). In summary, cow’s milk contains a variety of components that are known to promote immune homeostasis and induce regulatory responses by human immune cells *in vitro* (Figure 1). Therefore, we hypothesize that these immune regulatory effects aid in tolerance induction toward allergens or suppress immune responses in the upper airways that could aid in the protection against asthma exacerbation.

## Systemic Responses; the Gut–Lung Axis

**Figure 2:**
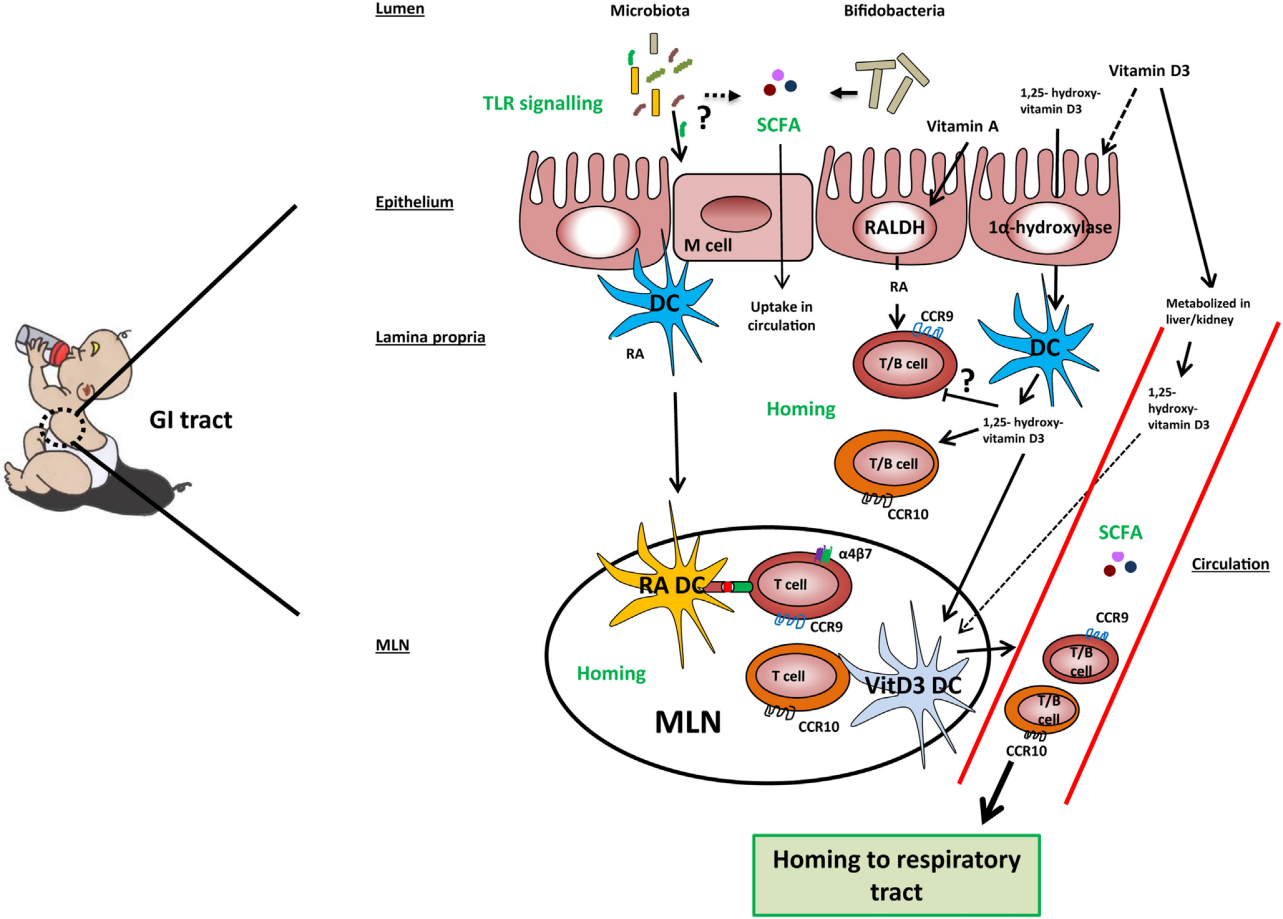
Mechanisms involved in the gut-lung axis linked to milk components. TLR signaling by microbiota in the gut results in improved airway immunity although the exact mechanism in this gut–lung axis is unknown. Microbiota, specifically Bifidobacteria, may ferment sialylated oligosaccharides present in cow’s milk into short-chain fatty acids (SCFAs), which are taken up into the circulation. Cow’s milk contains vitamin A, vitamin D3, and 1,25-hydroxyvitamin D3. Vitamin A and vitamin D3 can be taken up by epithelial and dendritic cells (DCs) and converted by the enzymes RALDH and 1α-hydroxylase into retinoic acid (RA) and 1,25-hydroxyvitamin D3, respectively. However, the majority of vitamin D3 is taken up into the systemic and converted into its active form in the kidneys. In the lamina propria, RA may induce expression of gut homing markers CCR9 and α4β7 on T cells and CCR9 on B cells directly or prime DC to induce the expression of these markers in the mesenteric lymph node (MLN). 1,25-hydroxyvitamin D3 may induce the expression of homing marker CCR10 on T and B cells directly or indirectly *via* vitamin D3-primed DC (VitD3 DC). In addition, 1,25-hydroxyvitamin D3 downregulates the expression of CCR9 on lymphocytes in a direct way. Lymphocytes expressing CCR9 have a homing capacity toward the small intestine, while lymphocytes expressing CCR10 have a homing potential toward the colon and respiratory tract, providing a potential mechanism of the gut–lung axis.

Several recent studies have shown that immune responses triggered in the GI tract can influence immunity in the respiratory tract. Evidence for the existence of this so-called gut–lung axis is increasing, although the exact mechanisms involved are not yet completely understood (125–128). The importance of TLR signaling by commensal microbiota in relation to airway immunity was demonstrated in multiple studies (129–131). Mice treated with antibiotics before influenza infection showed a higher viral load in the lungs, reduced CD4^+^ T cells responses and reduced influenza specific antibody titers compared to control mice. Intrarectal administration of TLR agonists could restore immune responses to influenza infection in this model. To clear the influenza infection, commensal bacteria or TLR agonists were needed to induce inflammasome-dependent cytokine release (IL-1β and IL-18). These cytokines allowed lung DCs to migrate to the mediastinal lymph node where they activate specific T cells (129). Another study in mice showed that oral administration of a bacterial extract (OM-85) reduced the viral load in the respiratory tract after influenza infection. The bacterial extract also boosted specific polyclonal antibodies against *Klebsiella pneumoniae* and *Streptococcus pneumoniae*, which protected the mice against these airway pathogens (130). Furthermore, germ-free mice showed increased susceptibility to pulmonary infection with *K. pneumonia*e, which could be restored by i.p. injection of LPS (131). These studies indicate that there is cross-talk between the commensal microbiota and immunity in the respiratory tract *via* TLR signaling (Figure 2).

Another link between airway immunity and the gut microbiota is the release of SCFAs. SCFAs are metabolites produced by bacteria in the gut from dietary non-digestible fibers. One type of non-digestible fibers are sialylated oligosaccharides present in cow’s milk. As mentioned earlier, milk oligosaccharides are fermented in the colon into SCFAs (51). *B. longum infantis* has been shown to ferment sialylated oligosaccharides directly (52, 53). After release into the colon, SCFAs such as acetate and to a lesser extent propionate are taken up into the circulation in mice (132) and humans (133). SCFAs bind to metabolite-sensing G protein-coupled receptors, and signaling influences gene expression *via* induction of histone deacetylases (133). Both acetate and propionate bind *via* GPR41, which is expressed on various tissues and cells including enteroendocrine cells and PBMCs (134). Several studies indicate that SCFAs play an important role in the gut–lung axis by regulating immune activation in the lung (132, 133) (Figure 2). A high-fiber diet was prevented against allergic airway disease (AAD) in mice (132, 133). This protective effect was shown to be mediated by acetate produced by the microbiota. Direct oral administration of acetate resulted in higher Treg numbers in the lung and protection against HDM-induced AAD (133). In another study, oral administered propionate did not affect Treg numbers in the lung, but resulted in hematopoiesis in the bone marrow of DCs that were found in the lungs. These DCs had a more immature phenotype (lower levels of MHCII and CD40) and therefore a reduced capacity of activating Th2 cells (132). These studies demonstrate that microbial metabolites produced in the intestines can have an effect on immune function in the airways. In addition, other microbial components such as TLR ligands may be taken up in the circulation and impact immunity in the respiratory tract. TLR stimulation in the gut could activate DCs leading to the activation of lymphocytes in the mediastinal lymph node. Upon activation, these lymphocytes can migrate to the lung and potentially to the gut. Besides, microbiota can have an indirect effect *via* SCFA production, as SCFAs in the circulation can affect DCs and Tregs in the respiratory tract. Currently, direct effects of SCFAs on the induction of homing markers on DCs or lymphocytes are not known.

After activation, lymphocytes can migrate (i.e., home) to tissues depending on their homing marker (e.g., selectins, integrins, and chemokine receptors) expression. These receptors can bind to tissue-specific ligands (e.g., addressins and chemokines) expressed by the endothelium. In humans, mucosal vaccination was used as a model to show that the site of induction of a mucosal immune response resulted in IgA production in restricted mucosal tissues. Holmgren and Czerkinsky showed that specific IgA antibodies are produced in the upper respiratory tract and gut in cholera toxin B (CTB) vaccinated individuals. In contrast, intranasal vaccination with CTB resulted in specific IgA production in both upper and lower respiratory tract and genital tract, but not in the gut. Furthermore, rectally vaccinated individuals only produced specific IgA locally in the rectum (135). The fact that orally administered antigens result in effector cells being present both in the gut and the upper respiratory tract indicates that homing markers might overlap. Well-studied homing marker interactions in humans are among others, CCR9 binding to locally produced CCL25 in the small intestine (136, 137) and CCR10 binding to CCL28 produced in the airways and colon (138). For B cells, there is no clear homing marker that differentiates between upper and lower respiratory tract homing as CCR10 expressed on B cells binds CCL28 produced locally in lower and upper respiratory tracts. In contrast, T cells express CCR10 to bind CCL28 produced in the lower respiratory tract and salivary glands (16, 139), while T cells express CCR3 to bind CCL28 in the nasal mucosal (140). Another homing marker that could be important in migration between gut and lung is CCR6 as its ligand CCL20, which are expressed in both tissues (141).

Thus, the homing potential of immune cells is affected by the site of induction and is dependent on local production of tissue-specific stromal factors. Recent evidence suggests that it can also be modified by dietary components. Of all dietary components, the effect on homing is best studied for RA and 1,25-dihydroxyvitamin D3 (Figure 2). Dietary vitamin A as a source of RA is essential for efficient homing of T cells to the GALT (142, 143). In mice, RA production by mucosal CD103^+^ DCs (143, 144) or stromal cells in the MLN (145) is essential for efficient differentiation of naive T cells into FoxP3^+^ Tregs that express the gut homing markers α4β7 and CCR9 in the MLN. Interestingly, human RA-primed CD103^+^ DCs were also shown to induce differentiation of naive T cells into IL-10-producing T cells expressing gut homing markers *in vitro* (122). RA is also a factor that regulates B cell proliferation, differentiation, and class switching (146). Moreover, RA and TGFβ1 induce IgA class switching (147). Similarly to the effects observed on T cells, RA derived from GALT-DCs alone was shown to induce gut homing markers on B cells (148). Interestingly, vitamin D3 blocks the upregulation of RA-induced gut homing marker expression on T cells (149, 150) although this was not observed by Baeke et al. (151). The majority of dietary vitamin D3 is taken up along the GI tract and converted into its active form 1,25(OH)2D3 in the kidney and becomes systemically available (94). In addition, vitamin D3 is shown to be converted in its active metabolite by DCs and epithelial cells (93, 149). Dietary supplementation of vitamin D3 to HIV-infected patients was shown to induce CCR10 expression on Tregs (152). This finding is in line with *in vitro* studies showing that vitamin D3 induces CCR10 expression on human B and T cells (149, 151, 153). Interestingly, RA also induces CCR10 expression in human B cells and acts even synergistically with 1,25-dihydroxyvitamin D3 (153). The balance of vitamin A and vitamin D3 may thereby regulate homing of lymphocytes to gut or respiratory tract, respectively (Figure 2). However, it should be noted that the concentrations of vitamin A and vitamin D3 are relatively low in cow’s and breast milk. In addition, the active metabolites of these vitamins can be endogenously produced (e.g., by stromal cells) in the secondary lymphoid tissues. In summary, we are only beginning to unravel the complex interplay between gut and lung. Hence, we can only speculate about the mechanisms by which cow’s milk through sialylated oligosaccharides and vitamin A and D could affect microbiota composition or homing of lymphocytes, respectively.

## Concluding Remarks

The existing epidemiological evidence suggests that the consumption of raw cow’s milk contributes to protection against allergies and asthma and respiratory tract infections. In this review, we discussed potential mechanisms by which cow’s milk and its components may exert these immunological effects. Bovine IgG can bind to bacterial and viral pathogens, enhance phagocytosis, and may neutralize pathogens. Other milk components like TGFβ promote epithelial barrier functioning by upregulation of TJ genes and might favor the differentiation of Tregs that can reduce inflammation locally. Finally, recent evidence show an interplay between gut and lung. We speculate about the effect of milk components on trafficking of lymphocytes from the intestine to the upper airways through modulation of homing receptors and microbiota. Further unraveling the impact of milk components on local responses in the respiratory tract, microbiota and immune trafficking are necessary to fully understand their effects on allergy, infection, and asthma.

## Author Contributions

All authors contributed to the writing process, prepared the manuscript, and approved the final version.

## Conflict of Interest Statement

RN is an employee of FrieslandCampina and MS received research funding from FrieslandCampina. All other authors declare that the research was conducted in the absence of any commercial or financial relationships that could be construed as a potential conflict of interest.
